# An updated classification of the Brassicaceae (Cruciferae)

**DOI:** 10.3897/phytokeys.220.97724

**Published:** 2023-03-06

**Authors:** Dmitry A. German, Kasper P. Hendriks, Marcus A. Koch, Frederic Lens, Martin A. Lysak, C. Donovan Bailey, Klaus Mummenhoff, Ihsan A. Al-Shehbaz

**Affiliations:** 1 South-Siberian Botanical Garden, Altai State University, Lenin Ave. 61, 656049 Barnaul, Russia Altai State University Barnaul Russia; 2 Department of Biology, Botany, University of Osnabrück, Barbarastraße 11, 49076 Osnabrück, Germany University of Osnabrück Osnabrück Germany; 3 Naturalis Biodiversity Center, Darwinweg 2, 2333 CR Leiden, Netherlands Naturalis Biodiversity Center Leiden Netherlands; 4 Department of Biodiversity and Plant Systematics, Centre for Organismal Studies (COS), Heidelberg University, Im Neuenheimer Feld 345, D-69120 Heidelberg, Germany Heidelberg University Heidelberg Germany; 5 Institute of Biology Leiden, Leiden University, Sylviusweg 72, 2333 BE Leiden, Netherlands Leiden University Leiden Netherlands; 6 Central European Institute of Technology (CEITEC) and Faculty of Science, Masaryk University, Kamenice 5, 62500 Brno, Czech Republic Masaryk University Brno Czech Republic; 7 Department of Biology, New Mexico State University, P.O. Box 30001 MSC 3AF, Las Cruces, NM 88003, USA New Mexico State University Las Cruces United States of America; 8 Missouri Botanical Garden, 4344 Shaw Boulevard, St. Louis, Missouri 63110, USA Missouri Botanical Garden St. Louis United States of America

**Keywords:** classification, subfamily, supertribe, taxonomy, tribe

## Abstract

Based on recent achievements in phylogenetic studies of the Brassicaceae, a novel infrafamilial classification is proposed that includes major improvements at the subfamilial and supertribal levels. Herein, the family is subdivided into two subfamilies, Aethionemoideae (subfam. nov.) and Brassicoideae. The Brassicoideae, with 57 of the 58 tribes of Brassicaceae, are further partitioned into five supertribes, including the previously recognized Brassicodae and the newly established Arabodae, Camelinodae, Heliophilodae, and Hesperodae. Additional tribus-level contributions include descriptions of the newly recognized Arabidopsideae, Asperuginoideae, Hemilophieae, Schrenkielleae, and resurrection of the Chamireae and Subularieae. Further detailed comments on 17 tribes in need of clarifications are provided.

## Introduction

Rapid advances in our understanding of phylogenetic relationships among taxa are driving the development of modern classification schemes that accurately reflect current knowledge. Brassicaceae (Cruciferae) is a relatively large family, currently comprising ca. 4140 species (original data), for which various classification systems have been proposed, including influential historical classifications contributed by de Candolle (1821), Hayek (1911), Schulz (1936), and Janchen (1942). The first infrafamilial classification for the Brassicaceae based on molecular phylogenetic data, proposed by Al-Shehbaz et al. (2006), included 25 tribes but no higher taxonomic units. The phylogenetic findings available at the time were based on relatively few species (e.g., ~ 100 spp.) and lacked clarity regarding the limits and relationships among the inferred major lineages (referred as to I, II, and III by Beilstein et al. 2006). Since then, numerous additional taxa have been included in phylogenetic studies and the amount, quality, and reliability of phylogenetic data has increased tremendously. This has led to the discussion of numerous informal evolutionary lineages (Huang et al. 2016; Nikolov et al. 2019) and the recognition of more than 50 tribes (e.g., Hohmann et al. 2015; Huang et al. 2020). Hence, there is an obvious need to codify the current well-supported understanding of Brassicaceae relationships (e.g., Nikolov et al. (2019), Walden et al. (2020), and especially Hendriks et al. (2022)) into an updated classification scheme that can now include robust subfamilial and supertribal groups.

## Taxonomy

### Brassicaceae

Taxon classificationPlantaeBrassicalesBrassicaceae

Burnett, Outlines Bot.: 854, 1093, 1123. Feb 1835, nom. cons., nom. alt.; Cruciferae Juss., Gen. Pl.: 237. 4 Aug 1789 nom. cons.

0CA51561-9A24-5DEE-91A0-2506F066B17A

#### Type.

*Brassica* L.

#### Distribution.

Cosmopolitan, centered in temperate regions of the Northern Hemisphere.

##### 1. Subfamilial division

All phylogenetic studies over the past two and a half decades identify *Aethionema* W.T. Aiton as sister to all other Brassicaceae, which supports the recognition of two highly unequal subfamilies, the new unigeneric Aethionemoideae with 58 species and the much bigger Brassicoideae, comprising the other 98.6% of species and the rest of the generic and tribal diversity of the family.

### Aethionemoideae

Taxon classificationPlantaeBrassicalesBrassicaceae

D.A.German, Hendriks, M.Koch, F.Lens, Lysak, C.D.Bailey, Mumm. & Al-Shehbaz subfam. nov.

88E15B95-34CE-56CE-9A7D-0806E3C668BC

urn:lsid:ipni.org:names:77315165-1

#### Type.

*Aethionema* W.T. Aiton

#### Description.

Trichomes and multicellular glands absent. Leaves entire, articulate at base. Fruits silicles, angustiseptate, bilocular, few-seeded, dehiscent, or unilocular, one-seeded, indehiscent; sometimes both types present. Most common *x* = 11, 12.

#### Distribution.

Primarily SW Asia, especially Turkey, Iran & Transcaucasia.

#### Tribes.

Aethionemeae Al-Shehbaz, Beilstein & E.A. Kellogg.

#### Note.

For many species of *Aethionema* a 3-nerved petal claw has been described (e.g., Hedge 1965). Further studies are needed to verify whether this is a feature present in all members of *Aethionema* and whether it is unique to the genus (and then diagnostic for the subfamily).

### Brassicoideae

Taxon classificationPlantaeBrassicalesBrassicaceae

Prantl, Text-book Bot.: 255. 1880 (‘Brassiceae’).

1D69A0A1-14A1-5F68-95EE-6BAB0790EECA

#### Type.

*Brassica* L.

#### Description.

Trichomes (simple and/or variously branched) and multicellular glands absent or present. Leaves entire to variously dissected, simple or compound, not articulate at base. Fruits various in compression, dehiscence, length to width ratio, number of seeds (one to > 100), etc. Base chromosome numbers various; the lowest *x* = 4.

#### Distribution.

Same as the whole family.

##### 2. Supertribal division

Brassicoideae is subdivided into the following five supertribes corresponding to the main evolutionary lineages discussed in detail by Hendriks et al. (2022).

### Arabodae

Taxon classificationPlantaeBrassicalesBrassicaceae

D.A.German, Hendriks, M.Koch, F.Lens, Lysak, C.D.Bailey, Mumm. & Al-Shehbaz, supertrib. nov.

84FBA7C5-2BA3-595B-A2E2-897C5B85D369

urn:lsid:ipni.org:names:77315210-1

#### Type.

*Arabis* L.

#### Description.

Trichomes present, mainly branched (exclusively or in combination with simple); multicellular glands absent. Leaves predominantly undivided or slightly divided, auriculate at base or not. Most common *x* = 8.

#### Distribution.

Mainly Northern Hemisphere (predominantly Holarctis of Eurasia, also of N America and Africa), S America (Andes).

#### Tribes.

Arabideae DC., Alysseae DC., Asperuginoideae trib. nov., Stevenieae Al-Shehbaz, D.A.German & M.Koch.

#### Notes.

Corresponds to evolutionary lineage IV of Nikolov et al. (2019) and Hendriks et al. (2022) or lineage D of Huang et al. (2016). Limits of this supertribe are not yet fully understood due to discordance in positions of tribes and their taxa in the nuclear vs. plastid phylogenies of Hendriks et al. (2022). It might be eventually restricted to Arabideae, while Alysseae and possibly Asperuginoideae would better be recognized as a separate supertribe, Alyssodae. Proper placement of Stevenieae also needs further clarification due to its grouping within Camelinodae lineage in chloroplast phylogenies (Walden et al. 2020; Hendriks et al. 2022).

### Brassicodae

Taxon classificationPlantaeBrassicalesBrassicaceae

V.E. Avet. in Biol. Zhurn. Armenii 43: 602. 1990 (‘Brassicidinae’).

82F9181A-55E0-5285-9ED9-E71524A977F5

#### Type.

*Brassica* L.

#### Syn.

Sisymbriodae V.E. Avet., Thelypodiodae V.E. Avet.

#### Description.

Trichomes absent or simple, rarely branched; multicellular glands absent. Leaves predominantly undivided or slightly divided, rarely much divided, often auriculate at base. Most common *x* = 7.

#### Distribution.

Mainly Northern Hemisphere (Holarctis of Eurasia, N America and Africa), to a lesser degree C and S America.

#### Tribes.

Aphragmeae D.A.German & Al-Shehbaz, Brassiceae DC. [incl. Bivonaeeae M.A. Koch & Warwick], Calepineae Horan., Coluteocarpeae V.I. Dorof., Conringieae D.A. German & Al-Shehbaz, Eutremeae Al-Shehbaz, Beilstein & E.A. Kellogg, Fourraeeae Al-Shehbaz, M.A. Koch, R. Karl & D.A.German, Isatideae DC., Kernereae Al-Shehbaz, Warwick, Mumm. & M.A. Koch, Plagiolobeae Khosravi & Eslami-Farouji, Schrenkielleae trib. nov., Sisymbrieae DC., Thelypodieae Prantl, Thlaspideae DC.

#### Notes.

Corresponds to evolutionary lineage II introduced by Beilstein et al. (2006) and subsequently modified by Franzke et al. (2011) to become known as “expanded lineage II”, or lineage B of Huang et al. (2016). Cochlearieae reveals relationship with Brassicodae in nuclear-based phylogeny, though it groups with “rogue” tribes of Heliophilodae in plastid trees (details in Hendriks et al. 2022). Its supertribal assignment is therefore yet unclear.

### Camelinodae

Taxon classificationPlantaeBrassicalesBrassicaceae

D.A.German, Hendriks, M.Koch, F.Lens, Lysak, C.D.Bailey, Mumm. & Al-Shehbaz, supertrib. nov.

C4F9117F-0CE7-5FA1-AF93-0C03D419A17B

urn:lsid:ipni.org:names:77315211-1

#### Type.

*Camelina* Crantz

#### Description.

Trichomes usually present, simple and/or branched; multicellular glands absent. Leaves not or variously divided to compound, auriculate at base or not. Base numbers various, most common *x* = 6, 7, 8.

#### Distribution.

Represented by native taxa at all continents except Antarctica; most diverse in Holarctis of Eurasia and N America.

#### Tribes.

Alyssopsideae Al-Shehbaz, Warwick, Mumm. & M.A. Koch, Arabidopsideae trib. nov., Boechereae Al-Shehbaz, Beilstein & E.A. Kellogg, Camelineae DC., Cardamineae Dumort., Crucihimalayeae D.A.German & Al-Shehbaz, Descurainieae Al-Shehbaz, Beilstein & E.A. Kellogg, Erysimeae Dumort., Halimolobeae Al-Shehbaz, Beilstein & E.A. Kellogg, Hemilophieae trib. nov., Lepidieae DC., Malcolmieae Al-Shehbaz & Warwick, Microlepidieae Al-Shehbaz, Warwick, Mumm. & M.A. Koch, Oreophytoneae Al-Shehbaz, Warwick, Mumm. & M.A. Koch, Physarieae B.L. Rob., Smelowskieae Al-Shehbaz, Beilstein & E.A. Kellogg, Turritideae Buchenau, Yinshanieae Al-Shehbaz, Warwick, Mumm. & M.A. Koch.

#### Notes.

Corresponds to evolutionary lineage I of Beilstein et al. (2006) and subsequent studies, or lineage A of Huang et al. (2016). Two genera of Camelinodae, *Chrysochamela* Boiss. and *Pseudoarabidopsis* Al-Shehbaz, O’Kane & R.A. Price, both excluded from Camelineae (see discussion below) are currently unassigned to a tribe.

### Heliophilodae

Taxon classificationPlantaeBrassicalesBrassicaceae

D.A.German, Hendriks, M.Koch, F.Lens, Lysak, C.D.Bailey, Mumm. & Al-Shehbaz, supertrib. nov.

F0209FFE-A49A-59B5-B9B7-CE167C1A8BC0

urn:lsid:ipni.org:names:77315212-1

#### Type.

*Heliophila* L.

#### Description.

Trichomes absent or simple, rarely branched; multicellular glands absent. Leaves mainly not or slightly divided, rarely much divided to compound, usually not auriculate at base. Base numbers are various due to post-polyploid diploidization – 12 tribes have originated through whole-genome duplications (data lacking for Hillielleae).

#### Distribution.

Well-represented in both Hemispheres; Eurasia (mainly SW Asia & S Europe), N, Tropical & S Africa, C & S America, New Zealand.

#### Tribes.

Anastaticeae DC., Asteae Al-Shehbaz, Warwick, Mumm. & M.A. Koch [incl. Scoliaxoneae Al-Shehbaz & Warwick], Biscutelleae Dumort., Chamireae Sond., Cremolobeae R. Br., Eudemeae Al-Shehbaz, Warwick, Mumm. & M.A. Koch, Heliophileae DC., Hillielleae H.L. Chen, T. Deng, J.P. Yue, Al-Shehbaz & H. Sun, Iberideae Webb & Berthel., Megacarpaeeae Kamelin ex D.A.German, Notothlaspideae Al-Shehbaz, Warwick, Mumm. & M.A. Koch, Schizopetaleae R. Br. ex Barnéoud, Subularieae DC.

#### Notes.

This group corresponds to evolutionary lineage V of Nikolov et al. (2019) and Hendriks et al. (2022). Anastaticeae, Biscutelleae, Hillielleae, Iberideae, and Megacarpaeeae are tentatively assigned to Heliophilodae due to their partially resolved phylogenetic position (grouping with others only in nuclear-based trees; see Hendriks et al. (2022) for details and discussion). Eventually, these five tribes may be recognized as a separate supertribe, e.g., Anastaticodae, based on the most speciose tribe among them. In the latter case, Heliophilodae would become unique among supertribes being almost completely restricted to the Southern Hemisphere.

### Hesperodae

Taxon classificationPlantaeBrassicalesBrassicaceae

D.A.German, Hendriks, M.Koch, F.Lens, Lysak, C.D.Bailey, Mumm. & Al-Shehbaz, supertrib. nov.

2D68C40A-6454-529D-8387-0EF1186281A0

urn:lsid:ipni.org:names:77315213-1

#### Type.

*Hesperis* L.

#### Description.

Trichomes usually present, simple and/or branched; multicellular glands often present. Leaves normally little divided, nearly never auriculate at base. Most common *x* = 7.

#### Distribution.

Native to Eurasia (predominantly temperate and dry subtropical Asia).

#### Tribes.

Anchonieae DC., Buniadeae DC., Chorisporeae C.A. Mey., Dontostemoneae Al-Shehbaz & Warwick, Euclidieae DC., Hesperideae Prantl, Shehbazieae D.A.German.

#### Note.

Corresponds to evolutionary lineage III of Beilstein et al. (2006) and subsequent studies, or lineage E of Huang et al. (2016).

##### 3. New tribal adjustments

Updates at the tribal level include recognition of additional six tribes, of which four are newly described and another two are resurrected. Tribal names are followed in parenthesis by numbers of genera and species.

###### 3a. Tribal assignment of *Arabidopsis*

Huang et al. (2016) were the first to show that *Arabidopsisthaliana* (L.) Heynh. and *A.lyrata* (L.) O’Kane & Al-Shehbaz formed a clade unrelated to the core Camelineae representatives *Capsellarubella* Reut., *Catolobuspendulus* (L.) Al-Shehbaz, and *Camelinasativa* (L.) Crantz. Nikolov et al. (2019) obtained the same results using the same taxa minus *Catolobus* (C.A. Mey.) Al-Shehbaz. Their findings are fully supported by Hendriks et al. (2022). As a result, *Arabidopsis* (DC.) Heynh. is placed in its own tribe.

### Arabidopsideae

Taxon classificationPlantaeBrassicalesBrassicaceae

Al-Shehbaz, Hendriks, M.Koch, F.Lens, Lysak, C.D.Bailey, Mumm. & D.A.German, trib. nov. (1: 18)

FDF33C61-51BB-515A-A6A2-34F9E01084DB

urn:lsid:ipni.org:names:77315214-1

#### Type.

*Arabidopsis* (DC.) Heynh.

#### Description.

Herbs, annual or perennial. Trichomes simple, mixed with stalked 1–3(or 4)-forked. Multicellular glands absent. Cauline leaves petiolate to subsessile and cuneate to attenuate at base, not auriculate. Racemes ebracteate, often elongated in fruit. Flowers actinomorphic; sepals ascending to spreading, base of lateral pair slightly saccate or not; petals white, pink, or purple; claw obscurely differentiated from blade or distinct; filaments unappendaged, wingless; pollen 3-colpate; ovules 15–80 per ovary. Fruits siliques, linear, terete or latiseptate, unsegmented; styles obsolete or to 1 mm long; stigma entire. Seeds uniseriate; cotyledons accumbent or rarely incumbent. *x* = 5 and 8.

#### Distribution.

Eurasia, Africa, North America.

#### Notes.

Arabidopsideae is distinguished from the Camelineae by the lack of stellate and dendritic trichomes, though both also have simple and stalked forked trichomes, by having petiolate or subsessile cauline leaves not auriculate at base, by the lack of yellow flowers, 15–80 ovules per ovary, silique fruits, and accumbent or rarely incumbent cotyledons. By contrast, the Camelineae usually have some stellate or dendritic trichomes, always sessile and auriculate to sagittate cauline leaves, usually yellow flowers, though white to pink flowers occur just as in the Arabidopsideae, 2–40 ovules per ovary, silicle or rarely silique fruits, and incumbent or rarely accumbent cotyledons.

##### 3b. *Asperuginoides*

There has been no agreement among various authors about the tribal assignment of monospecific *Asperuginoides* Rauschert. For example, Khosravi et al. (2009) indicated a close relationship to the Cochlearieae, whereas German et al. (2009) and Warwick et al. (2010) showed no affinity to any tribe. It was listed as an unplaced genus by Al-Shehbaz (2012). More recently, Nikolov et al. (2019) and Hendriks et al. (2022) identified a sister relationship to the Alysseae, but Španiel et al. (2015) excluded it from the tribe. Furthermore, the plastome data by Walden et al. (2020) did not support that nor indicated any relationship to the 50+ tribes. Given the current data, it appears that the best solution is to place this anomalous genus in its own tribe.

### Asperuginoideae

Taxon classificationPlantaeBrassicalesBrassicaceae

Al-Shehbaz, Hendriks, M.Koch, F.Lens, Lysak, C.D.Bailey, Mumm. & D.A.German, trib. nov. (1: 1)

B6F84E5D-77DC-5A0A-A0DF-1F18E3170D7B

urn:lsid:ipni.org:names:77315215-1

#### Type.

*Asperuginoides* Rauschert

#### Description.

Herbs annual. Trichomes stalked, stellate or substellate, 4–6-rayed, these mixed with glochidate ones on fruit. Multicellular glands absent. Cauline leaves petiolate, not auriculate. Racemes bracteate throughout, usually elongated in fruit, with strongly recurved fruiting pedicels. Flowers actinomorphic; sepals ascending, base of lateral pair not saccate; petals white, claw undifferentiated from blade; filaments slender at base, unappendaged; pollen 3-colpate; ovules 2 per ovary, apical. Fruits dehiscent silicles, suborbicular, latiseptate, unsegmented, wingless, with long-stalked, setose, stiff trichomes glochidiate at apex; septum complete or absent; style distinct; stigma entire. Seeds aseriate, broadly winged; cotyledons accumbent. *x* = 16.

#### Distribution.

Afghanistan, Armenia, Iran, Kazakhstan, Kyrgyzstan, Pakistan, Tajikistan, Turkey, Turkmenistan, Uzbekistan.

##### 3c. *Chamira*

Although the tribe Chamireae was first recognized by Sonder (1846) and later accepted by Schulz (1936), it has not been widely recognized since, and *Chamira* Thunb. was listed as unplaced in Al-Shehbaz (2012). The findings of Hendriks et al. (2022) agree with those of Mummenhoff et al. (2005), Mandáková et al. (2012), Nikolov et al. (2019), Walden et al. (2020), and Dogan et al. (2021) that *Chamira* and *Heliophila* are closely related genera that do not belong to the same tribe, and the former has been used as the outgroup for phylogenetic and genomic studies of the latter. A tribal description comparable to that of other tribes is provided below.

### Chamireae

Taxon classificationPlantaeBrassicalesBrassicaceae

Sond. in Abh. Naturwiss. Verein Hamburg 1: 267. 1846. (1: 1)

ECC2384A-A4A3-5601-8AA4-4291A0E9B181

#### Type.

*Chamira* Thunb.

#### Description.

Herbs, annual. Trichomes absent. Leaves sessile or short petiolate, not auriculate at base, lowest pair opposite, representing persistent cotyledons and main photosynthetic part of plant, to 25 cm wide, cauline leaves alternate, much smaller, sometimes fail to develop. Racemes ebracteate, elongated in fruit. Sepals connivent, dimorphic, median (outer) pair not saccate at base, lateral pair with a distinct spur 1–2.5 mm long; petals white, with well-differentiated claw; filaments unappendaged; pollen 3-colpate; ovules 2–8 per ovary. Fruits siliques, dehiscent, terete to sublatiseptate, unsegmented; styles distinct; stigma entire. Seeds uniseriate; cotyledons longitudinally folded and margins deeply folded within. *x* = 19.

#### Distribution.

*Chamiracircaeoides* (L. f.) Zahlbr. is endemic to the Western Cape of South Africa.

##### 3d. *Dipoma* and *Hemilophia*

*Dipoma* Franch. was first studied by Warwick et al. (2010) who did not assign it to any tribe, and together with *Hemilophia* Franch., they were listed as unplaced in Al-Shehbaz (2012). Nikolov et al. (2019) showed the two genera form a monophyletic clade unrelated to any tribe and suggested their placement in a new tribe. However, plastome data by Walden et al. (2020) showed *Dipoma* to be affiliated with the Crucihimalayeae and not with *Hemilophia*. The results from the nuclear genome of Hendriks et al. (2022) fully agree with those of Nikolov et al. (2019), and the new tribe Hemilophieae is proposed here to accommodate both genera, leaving incongruent chloroplast and nuclear-based phylogenies.

### Hemilophieae

Taxon classificationPlantaeBrassicalesBrassicaceae

Al-Shehbaz, Hendriks, M.Koch, F.Lens, Lysak, C.D.Bailey, Mumm. & D.A.German, trib. nov. (2: 7)

0B80A346-B478-571F-B4F8-7E35106AF293

urn:lsid:ipni.org:names:77315216-1

#### Type.

*Hemilophia* Franch.

#### Description.

Herbs rhizomatous perennials. Trichomes simple, malpighiaceous, sometime short-stalked forked. Multicellular glands absent. Cauline leaves petiolate to subsessile and cuneate to attenuate at base, not auriculate. Racemes bracteate throughout, elongated or not in fruit. Flowers actinomorphic; sepals ascending to spreading, base of lateral pair not saccate; petals white, pink, or purple; claw obscurely differentiated from blade or distinct; filaments slender or dilated at base and sometimes strongly appendaged; pollen 3-colpate; ovules 2 or 4 per ovary, apical. Fruits dehiscent silicles, oblong to ovoid, terete or slightly angustiseptate, unsegmented, wingless or with narrow wings or crests; septum complete or absent; styles distinct, cylindrical or conical; stigma entire. Seeds aseriate; cotyledons accumbent. Base numbers various.

#### Distribution.

Endemic to China (Sichuan and Yunnan).

#### Note.

The tribe includes narrowly distributed monospecific *Dipoma* and *Hemilophia* (6 spp.).

##### 3e. *Idahoa* and *Subularia*

Beilstein et al. (2006, 2008) studied two samples of *Idahoa* A. Nelson & J.F. Macbr. and their position was unresolved in a polytomy that included *Asta* Klotzsch ex O.E. Schulz and Cremolobeae (*Cremolobus* DC. and *Menonvillea* DC.). Couvreur et al. (2010) sampled only *Subularia* L. but it was oddly placed in the Isatideae. By contrast, the family-wide phylogenetic study of Warwick et al. (2010) was the first that dealt with both *Idahoa* and *Subularia*. The former was sister clade to *Petrocallis* W.T. Aiton and together they were sister to *Subularia*. That clade was sister to many taxa of various tribes. These early studies did not resolve the relationship of both genera, and Al-Shehbaz (2012) listed both genera as unplaced.

The first clear relationship of *Idahoa* and *Subularia* to other tribes was given in Nikolov et al. (2019). The two genera formed a monophyletic group sister to a clade of *Asta* and *Scoliaxon* Payson (Asteae), which was sister to the South American CES clade of Salariato et al. (2016): *Cremolobus* (Cremolobeae), *Brayopsis* Gilg & Muschl. (Eudemeae), and *Schizopetalon* Sims (Schizopetaleae). The findings of Walden et al. (2020) and Dogan et al. (2022) were basically similar in terms of the entire complex of tribes except for minor differences in the position of *Idahoa* and *Subularia* relative to the other tribes. The findings of Hendriks et al. (2022) are basically the same except for the unexpected position of *Teesdalia* W.T. Aiton (Iberideae) between *Subularia* and *Idahoa*, and further studies should resolve such a relationship. Regardless of the slight differences in the most recent plastid vs. nuclear family-wide phylogenies, it is evident that these two genera should be placed in one tribe, and the name Subularieae was validly proposed over two centuries ago.

### Subularieae

Taxon classificationPlantaeBrassicalesBrassicaceae

DC., Mém. Mus. Hist. Nat. 7(1): 257. 20 Apr 1821. (2: 3)

510F50C1-E164-5C7C-BFB8-6026C291D180

#### Type.

*Subularia* L.

#### Description.

Herbs scapose annuals. Trichomes absent. Multicellular glands absent. All leaves in a basal rosette, sessile or petiolate, cauline leaves absent. Racemes ebracteate throughout and elongated or not in fruit, or flowers solitary on long pedicels originating from center of rosette. Flowers actinomorphic; sepals spreading or ascending, base of lateral pair not saccate; petals white, claw obscure or undifferentiated from blade; filaments slender at base; pollen 3-colpate; ovules 4–18. Fruits dehiscent, unsegmented silicles, orbicular and strongly latiseptate or obovoid to ellipsoid and slightly angustiseptate; septum complete; styles minute or absent; stigma entire. Seeds biseriate, broadly winged and accumbent, or wingless and incumbent. *x* = 14 and 15.

#### Distribution.

The tribe includes monospecific *Idahoa* (NW USA and Canadian British Columbia) and two aquatic or littoral species of *Subularia*, of which *S.monticola* A. Braun ex Schweinf. is restricted to tropical East Africa, and *S.aquatica* L. is distributed in northern North America (subsp. americana G.A. Mulligan & Calder) and temperate Eurasia (subsp. aquatica).

##### 3f. *Schrenkiella*

This monospecific genus was based on *Diplotaxisparvula* Schrenk, a species that fluctuated between unrelated genera solely on morphological grounds. It was first shown by German et al. (2009) to occupy an isolated position among Asian Brassicaceae and was subsequently recognized by German and Al-Shehbaz (2010) as a monospecific genus that was not placed in any tribe. It was shown by Huang et al. (2016) to form a basal clade to that including *Sisymbrium* L. and six genera of the Brassiceae. The first robust position of *Schrenkiella* was shown by Walden et al. (2020) and fully supported by Hendriks et al. (2022). It is sister to a clade including the Fourraeeae and sister clade including the Brassiceae and Isatideae plus Sisymbrieae and Thelypodieae. The isolated position of monophyletic *Schrenkiella* strongly supports its placement in its own tribe.

### Schrenkielleae

Taxon classificationPlantaeBrassicalesBrassicaceae

Al-Shehbaz, Hendriks, M.Koch, F.Lens, Lysak, C.D.Bailey, Mumm. & D.A.German, trib. nov. (1: 1)

BD9B44E5-D2D2-54E5-A6CD-EADFE348B879

urn:lsid:ipni.org:names:77315217-1

#### Type.

*Schrenkiella* D.A.German & Al-Shehbaz

#### Description.

Herbs annual, glaucous. Trichomes absent. Multicellular glands absent. Cauline leaves petiolate to subsessile, fleshy, cuneate at base, not auriculate. Racemes ebracteate, elongated in fruit, rachis strongly flexuous. Flowers actinomorphic; sepals suberect, base of lateral pair not saccate; petals absent, rarely present, white, subequaling sepals; claw obsolete; filaments slender, unappendaged; pollen 3-colpate; ovules 24–50 per ovary. Fruits dehiscent siliques, linear, latiseptate, unsegmented; septum complete; styles distinct; stigma entire. Seeds biseriate; cotyledons incumbent. *x* = 7.

#### Distribution.

*Schrenkiellaparvula* (Schrenk) D.A.German & Al-Shehbaz is sporadically distributed in Armenia, Azerbaijan, Iran, Kazakhstan, Russia, Turkey, Turkmenistan, and Uzbekistan.

##### 4. Further tribal comments

The following alphabetical tribal discussions are based on the phylogenies of Hendriks et al. (2022), along with comparison of the recent family-wide phylogenies of Nikolov et al. (2019), Walden et al. (2020), and few earlier ones. Generic limits and species number closely follow BrassiBase (Kiefer et al. 2014) with some updating. As above, tribal names are followed in parenthesis by numbers of genera and species, and those that showed no conflict with previous phylogenies are not discussed here. They include Anastaticeae (13: 67), Aphragmeae (1: 13), Biscutelleae (5: 74), Boechereae (9: 125), Buniadeae (1: 2), Calepineae (3: 9), Cardamineae (14: 344), Chorisporeae (4: 56), Cochlearieae (2: 29), Coluteocarpeae (1–12: 130), Crucihimalayeae (3: 15), Erysimeae (1: 274), Euclidieae (30: 155), Eutremeae (1: 44), Halimolobeae (5: 39), Heliophileae (1: 105), Hesperideae (1: 52), Isatideae (5: 99), Kernereae (3: 3), Lepidieae (1: 268), Malcolmieae (1: 6), Megacarpaeeae (2: 11), Microlepidieae (15: 57), Notothlaspideae (1: 3), Oreophytoneae (2: 7), Physarieae (7: 133), Schizopetaleae (4: 21), Shehbazieae (1: 1), Sisymbrieae (1: 49), Smelowskieae (1: 25), Stevenieae (2: 10), Thelypodieae (34: 235), Thlaspideae (13: 39), Turritideae (1: 2), and Yinshanieae (1: 4).

**Aethionemeae** (1: 58). The tribe is distributed primarily in SW Asia and the Mediterranean region, with the center of greatest diversity located in Turkey, in which 23 of the 40 species are endemic. All previous molecular studies have supported the tribal position as a sister clade to the rest of the Brassicaceae recognized above at subfamilial level.

**Alysseae** (24: 282). The tribe is almost exclusively distributed in Eurasia, with several native species in North Africa and one in North America. The largest and most complex genera are *Alyssum* L. and *Odontarrhena* C.A. Mey. ex Ledeb. with about 114 and 91 species, respectively. The tribe has recently been revised by Španiel et al. (2015), and its database AlyBase (www.alysseae.sav.sk; Španiel et al. 2015) should be consulted for further data and updates. All except *Brachypus* Ledeb. (1 sp.), *Galitzkya* V.V. Botschantz. (3 spp.), and *Takhtajaniella* V.E. Avet. (1 sp.) are included in the phylogeny by Hendriks et al. (2022).

**Alyssopsideae** (4: 9). A small Asian tribe distributed predominantly in Afghanistan, Azerbaijan, Iran, Tajikistan, and Turkmenistan. It is monophyletic in Hendriks et al. (2022) and a sister clade to *Chrysochamela* and together are sister to *Pseudoarabidopsis*. These two genera belong to paraphyletic Camelineae III and together are sister to the Turritideae (2 spp.). The sister relationship of *Pesudoarabidopsis* to the Turritideae was demonstrated earlier by Walden et al. (2020) who showed that their clade is distinct from the Camelineae including the generic type *Camelina*. It is clear that these taxa do not belong to the Camelineae s. str. (Hendriks et al. 2022), but further studies are needed to explore whether they are well supported within Alyssopsideae.

**Anchonieae** (9: 75). Except for several species of *Matthiola* W.T. Aiton in Europe, the tribe is distributed primarily in SW and C Asia, and Africa. Only monospecific *Eremoblastus* Botsch. is not covered in Hendriks et al. (2022). The generic type, *Anchonium* DC., has recently been reduced to synonymy of the earlier-published *Sterigmostemum* M. Bieb. (German and Al-Shehbaz 2017). The tribe is characterized by the presence of multicellular-multiseriate glands, though apparently these structures were independently lost in *Veselskya* Opiz (1 sp.), one species of *Sterigmostemum*, and some species of *Matthiola* (ca. 56 spp.). Such glands are also found in the related tribes Chorisporeae and Dontostemoneae.

**Arabideae** (18: 559). The tribe is the largest and most complex in the family. It includes ten monospecific genera, and *Draba* L. (ca. 410 spp.), *Arabis* L. (ca. 100 spp.), and *Aubrieta* Adans. (23 spp.) are the most species rich ones. The tribe has been the focal topic for the Koch lab (Heidelberg University) for about three decades and despite carving nearly a dozen segregates into several tribes, *Arabis* still needs further focus and taxonomic adjustments are under consideration (see Koch et al. 2022 for references).

**Asteae** (2: 2). The findings of Hendriks et al. (2022) strongly justify merging the Mexican monospecific tribe Scoliaxoneae with the earlier published Asteae. That clade is most closely related to the South American CES clade *sensu*Salariato et al. (2016). These findings are in full agreement with those of Walden et al. (2020), but not closely related to the European Kernereae, a tribe more closely related to the Cochlearieae, Conringieae, and Coluteocarpeae in Hendriks et al. (2022).

**Brassiceae** (53: 243). The tribe has been recognized by all authors since it was established by de Candolle (1821). With the exception of a few genera (e.g., *Ammosperma* Hook.f., *Bivonaea* DC., *Horwoodia* Turrill, and *Pseuderucaria* O.E. Schulz), the plants have conduplicate cotyledons and/or segmented (heteroarthrocarpous) fruits. All except four genera (*Cordylocarpus* Desf., *Fezia* Pit. ex Batt., *Muricaria* Desv., and *Rytidocarpus* Coss.) were included in Hendriks et al. (2022). Unlike the findings of Walden et al. (2020) based on chloroplast data, *Bivonaea* was placed as sister to the tribe Fourraeeae.

Monophyly of *Brassica* is established based on most recent molecular phylogenies (e.g., Hendriks et al. 2022). About a dozen species of *Brassica* have been transferred to *Guenthera* Andrz., but monophyly of the latter with additional species needs to be established. Two other genera of the tribe, *Diplotaxis* DC. and *Erucastrum* C. Presl, remain artificially delimited, and similar studies are needed to accurately define their boundaries.

**Camelineae** (4: 16). As shown by Hendriks et al. (2022), the Camelineae as hitherto accepted are paraphyletic, of which Camelineae I includes *Camelina* (8 spp.), *Capsella* Medik. (5 spp.), *Catolobus* (1 sp.), and *Neslia* Desv. (2 spp.). Camelineae III is discussed above in connection with the Alyssopsideae. Finally, Camelineae II includes only *Arabidopsis*, which is shown in Hendriks et al. (2022) and some earlier studies to form a distinct clade from the rest of the Camelineae and recognized above in its own tribe. With the exclusion of Camelineae II and III, the tribal description of Camelineae s.str. is updated below:

Herbs, annual or perennial. Trichomes stalked or sessile, stellate, dendritic, or forked, sometimes mixed with simple ones. Multicellular glands absent. Cauline leaves sessile, mostly entire, auriculate or sagittate at base. Racemes ebracteate, often elongated in fruit. Flowers actinomorphic; sepals erect to spreading, lateral pair often not saccate at base; petals white, yellow, orange, pink, or purple, often with a distinct claw; filaments unappendaged, wingless; pollen 3-colpate; ovules 2–40 per ovary. Fruits silicles or siliques, dehiscent or indehiscent, latiseptate, terete, or angustiseptate, unsegmented; styles often distinct; stigma entire or rarely 2-lobed. Seeds biseriate, uniseriate, or aseriate; cotyledons incumbent or rarely accumbent.

**Conringieae** (1: 3) vs. **Plagiolobeae** (1: 5). The Conringieae*sensu*Al-Shehbaz (2012) was broadly delimited to encompass a heterogenous assembly of the genera *Conringia* (6 spp.) and *Zuvanda* (3 spp.). The findings of Hendriks et al. (2022) agree with those of Walden et al. (2020) and Nikolov et al. (2019) in that the Conringieae s.l. is not monophyletic. Based on the molecular findings and re-evaluation of morphology in light of those studies, one species, *C.planisiliqua*, was assigned to the genus *Iljinskaea* (Al-Shehbaz et al. 2021) of the Isatideae, *Zuvanda* and three species of *Conringia* are currently recognized as five species of *Plagioloba* of the tribe Plagiolobeae (German 2021; German 2022; Khosravi et al. 2022), and the remaining three species of *Corningia* are retained in the genus. The Conringieae differs from the Plagiolobeae by having 4- to 8-angled (vs. terete) fruits and entire (vs. slightly to prominently 2-lobed) stigmas with connivent (or sometimes decurrent) lobes.

**Cremolobeae** (4: 32). As currently recognized (Salariato et al. 2016; Salariato et al. 2020), the tribe includes the genera *Aimara* Salariato & Al-Shehbaz (1 sp.), *Cremolobus* (5 spp.), *Menonvillea* (24 spp.), and *Yunkia* Salariato & Al-Shehbaz (2 spp.). Hendriks et al. (2022) included five species of the tribe that belong to the first three genera, and their findings support the monophyly of the tribe, as did the above studies of Salariato et al. (2016, 2020). However, Walden et al. (2020) showed that *Menonvillea* did not fall with the rest of the tribe, and further studies are definitely needed (see tribe Eudemeae below).

**Descurainieae** (6: 48). Except for the monospecific Patagonian *Trichotolinum* O.E. Schulz, which has not yet been included in any phylogenetic studies, the position of other five genera in Hendriks et al. (2022) agrees with earlier studies.

**Dontostemoneae** (2: 14). Position of Dontostemoneae, Chorisporeae, and their intertribal hybrid Shehbazieae are in full agreement with the initial findings by German and Friesen (2014) and Walden et al. (2020). In contrast to these consistent findings, Liu et al. (2021) probably erroneously considered *Shehbazia* D.A. German as member of the paraphyletic Chorisporeae.

**Eudemeae** (9: 40). Hendriks et al’s. (2022) sampling of five species of five genera supports the monophyly of this tribe. Together with the other exclusively South American tribes, Cremolobeae (see above) and Schizopetaleae of the CES clade *sensu*Salariato et al. (2016) and North American Asteae, the group forms a monophyletic New World clade. Such generic relationship was first observed by Walden et al. (2020) who demonstrated that *Menonvillea* falls outside the Cremolobeae. Salariato et al. (2022) showed that *Alshehbazia* Salariato & Zuloaga (3 spp.), *Aschersoniodoxa* Gilg & Muschl. (3 spp.), *Gongylis* Theophr. ex Molinari & Sánchez Och. (1 sp.), *Onuris* Phil. (5 spp.), and *Xerodraba* Skottsb. (5 spp.) are monophyletic, whereas *Brayopsis* (9 spp.), *Dactylocardamum* Al-Shehbaz (2 spp.), *Eudema* Humb. & Bonpl. (4 spp.), and *Stenodraba* O.E. Schulz (8 spp.) are polyphyletic. Clearly, the entire complex is much in need of further studies based on extensive sampling of most species of the entire complex.

**Fourraeeae** (2: 3) This tribe has recently been established by Koch et al. (2022) to accommodate three species previously assigned to *Arabis*. Those authors discussed previously published extensive molecular studies that did not support the placement of those species within *Arabis*. The group includes the European *Fourraeaalpina* (L.) Greuter & Burdet and two Moroccan species assigned to the new genus *Hurkaea* Al-Shehbaz, M.A. Koch, R. Karl & D.A. German. The data of Hendriks et al. (2022) strongly support the recognition of this tribe.

**Hillielleae** (1: 11). The recently established Hillielleae was previously part of the Yinshanieae, but Chen et al. (2016) clearly showed that the two tribes are distantly related. Walden et al. (2020) confirmed the findings of Chen et al. and demonstrated that the Hillielleae is sister to a clade containing the Iberideae and Megacarpaeeae but remotely related to the Biscutelleae. However, Hendriks et al. (2022) showed that the Hillielleae is sister to the clade including the last three tribes and together are sister to the Anastaticeae.

**Iberideae** (2: 30). The tribe includes the primarily European *Iberis* L. (27 spp.) and *Teesdalia* (3 spp.). Only Warwick et al. (2010) included *Teesdalia* in their studies and showed it to form a sister clade to *Iberis* and thus placed both genera in the tribe Iberideae. In Hendriks et al. (2022), two species of *Teesdalia* and one of *Iberis* were sampled and the results showed them to be remotely related. Clearly a better sampling of *Iberis* ought to be done to check whether or not the two genera can be maintained in one tribe.

## Concluding remarks

The taxonomic framework presented here reflects a growing body of phylogenetic knowledge derived from continual advances in the sampling of species, broader representation of major groups, and the extensive sampling of genomic regions needed to help robustly resolve relationships across scales (Hendriks et al. 2022). The consistent nature of those findings suggest that this classification is a considerable advance over previously available formal classifications. However, we are fully aware that further accumulation of phylogenetic data will result in additions and modifications to our understanding of relationships among a minority of Brassicaceae. Most importantly, elements of phylogenetic uncertainty, illustrated by the presence of a few “jumpy clades” and discordance between nuclear and plastid phylogenies, highlight both the need to continue to resolve Brassicaceae relationships and regions of “the family tree” that are likely to experience and require future taxonomic modifications.

## Supplementary Material

XML Treatment for Brassicaceae

XML Treatment for Aethionemoideae

XML Treatment for Brassicoideae

XML Treatment for Arabodae

XML Treatment for Brassicodae

XML Treatment for Camelinodae

XML Treatment for Heliophilodae

XML Treatment for Hesperodae

XML Treatment for Arabidopsideae

XML Treatment for Asperuginoideae

XML Treatment for Chamireae

XML Treatment for Hemilophieae

XML Treatment for Subularieae

XML Treatment for Schrenkielleae
